# Progressive multifocal leukoencephalopathy in a patient post allo-HCT successfully treated with JC virus specific donor lymphocytes

**DOI:** 10.1186/s12967-020-02337-5

**Published:** 2020-04-21

**Authors:** M. J. Steinhardt, E. Wiercinska, M. Pham, G. U. Grigoleit, A. Mazzoni, M. Da-Via, X. Zhou, K. Meckel, K. Nickel, J. Duell, F. C. Krummenast, S. Kraus, C. Hopkinson, B. Weissbrich, W. Müllges, G. Stoll, K. M. Kortüm, H. Einsele, H. Bonig, L. Rasche

**Affiliations:** 1 Department of Internal Medicine II, University Hospital Würzburg, Oberdürrbacher Street 6, 97080 Würzburg, Germany; 2 Department of Cellular Therapeutics (GMP), German Red Cross Blood Service BaWüHe, Institute Frankfurt, Frankfurt, Germany; 3 Institute of Diagnostic and Interventional Neuroradiology, University Hospital of Würzburg, Würzburg, Germany; 4 Immunohematology Unit, Azienda Ospedaliera Universitaria Pisana, Pisa, Italy; 5 Northeastern Oklahoma Community Health Center, Afton, OK USA; 6 Institute of Virology and Immunobiology, University of Würzburg, Würzburg, Germany; 7 Department of Neurology, University Hospital Würzburg, Würzburg, Germany; 8 Institute for Transfusion Medicine and Immunohematology, Goethe University, Frankfurt, Germany; 9 Mildred Scheel Early Career Center, University Hospital of Würzburg, Würzburg, Germany

**Keywords:** Myeloma, JCV, Prodigy, CCS, PML, Donor lymphocytes, Adaptive cell transfer

## Abstract

**Background:**

Progressive multifocal leukoencephalopathy is a demyelinating CNS disorder. Reactivation of John Cunningham virus leads to oligodendrocyte infection with lysis and consequent axonal loss due to demyelination. Patients usually present with confusion and seizures. Late diagnosis and lack of adequate therapy options persistently result in permanent impairment of brain functions. Due to profound T cell depletion, impairment of T-cell function and potent immunosuppressive factors, allogeneic hematopoietic cell transplantation recipients are at high risk for JCV reactivation. To date, PML is almost universally fatal when occurring after allo-HCT.

**Methods:**

To optimize therapy specificity, we enriched JCV specific T-cells out of the donor T-cell repertoire from the HLA-identical, anti-JCV-antibody positive family stem cell donor by unstimulated peripheral apheresis [1]. For this, we selected T cells responsive to five JCV peptide libraries via the Cytokine Capture System technology. It enables the enrichment of JCV specific T cells via identification of stimulus-induced interferon gamma secretion.

**Results:**

Despite low frequencies of responsive T cells, we succeeded in generating a product containing 20 000 JCV reactive T cells ready for patient infusion. The adoptive cell transfer was performed without complication. Consequently, the clinical course stabilized and the patient slowly went into remission of PML with JCV negative CSF and containment of PML lesion expansion.

**Conclusion:**

We report for the first time feasibility of generating T cells with possible anti-JCV activity from a seropositive family donor, a variation of virus specific T-cell therapies suitable for the post allo transplant setting. We also present the unusual case for successful treatment of PML after allo-HCT via virus specific T-cell therapy.

## Background

Progressive multifocal leukoencephalopathy (PML) is a frequently fatal CNS disorder caused by reactivation of JC virus (JCV). Virus replication in latently infected oligodendrocytes leads to axonal demyelination. Affected individuals frequently present with confusion or seizures but the clinical presentation mainly depends on the extent of demyelination and brain structures involved [2]. Due to profound T-cell depletion and potent immunosuppressive factors, allogeneic hematopoietic cell transplantation (allo-HCT) recipients are at risk for JCV reactivation. PML is almost universally fatal when occurring after allo-HCT [3] and new treatment approaches are highly warranted. Recently, PML attracted attention from the medical and scientific community as a first post allo-HCT patient apparently benefited from the adoptive transfer of allogeneic BK virus specific T-cells [4]. BK virus and JCV are genetically related and share a number of immunogenic epitopes. Prior to that, another group had already generated JCV specific T cells from a HLA matched donor. The adoptive transfer of these cells was well tolerated and the patient showed neurological improvement [5]. These two cases argue to the potential of T-cell transfers after allo-HCT, although only selected patients will be in a situation where T-cell transfer is an option.

Multiple myeloma (MM) patients treated with the anti-CD38 monoclonal antibody daratumumab (Dara) show an increased risk for infectious complications, potentially attributable to the depletion of NK cells [6–8]. Recently, reactivation of hepatitis B including fatalities was reported in Dara-treated patients suggesting a more complex immune suppression by CD38 antibodies also affecting T-cell function (Reactions Weekly (2019) 1747: 1. 10.1007/s40278-019-59642-3). CD38 is expressed on a subset of activated CD8^+^ T cells, which may explain a dysfunctional T-cell system in some Dara-treated patients. Here, we report the first case of PML after Dara-containing therapy during post-allogeneic transplant relapse. Remarkably, PML was successfully treated using multiple strategies, including the adoptive transfer of JCV specific donor lymphocytes.

## Case description

**Fig. 1:**
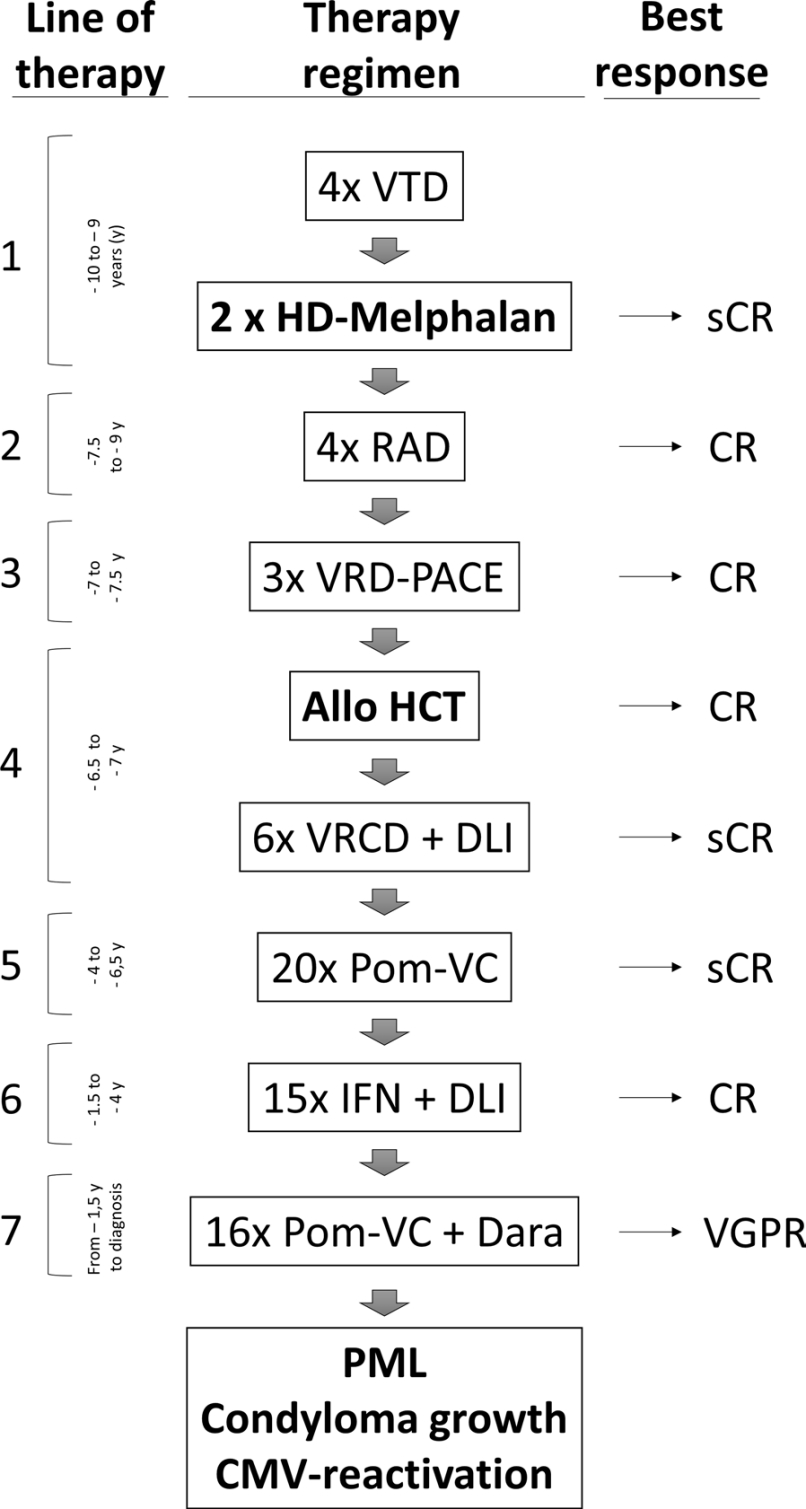
Previous MM therapy. Previous anti-MM therapy (middle row), their respective time intervals (left row) and disease responses (right row) before onset of PML. The patient received seven lines of therapy, including a tandem high dose (HD) melphalan therapy and an allo-HCT with successive interferon and donor lymphocyte infusions (DLI). The last line of therapy before JC virus reactivation contained Pomalidomide (Pom), Bortezomib, Cyclophosphamid and Daratumumab (Dara)

We describe the case of a 59-year old MM patient with an 11-year treatment history (Fig. 1). After failing to achieve a durable remission with seven previous lines of treatment, including an allo-HCT from an HLA-identical family donor 6.5 years earlier, he was currently undergoing his 20th cycle of Dara, pomalidomide, bortezomib, cyclophosphamide and dexamethasone. The patient developed seizures and a cerebral MRI revealed a posterior white matter lesion. This lesion showed a hyperintense T2 lesion in the left temporooccipital region with predominant involvement of the subcortical white matter with subsequent regional progression and increasing permeability of the blood–brain-barrier. Cerebrospinal fluid (CSF) analysis revealed JC virus reactivation with up to 560 copies/dL in two independent examinations. The constellation of clinical and radiological manifestations with JC virus positive CSF met the criteria for PML [9]. Notably, besides JC virus reactivation, the patient also presented with CMV reactivation, persistent parainfluenza type 3 positivity on throat swabs and human papilloma virus driven giant anal condyloma acuminatum, in line with severe disruption of T cell mediated immune surveillance.

**Fig. 2:**
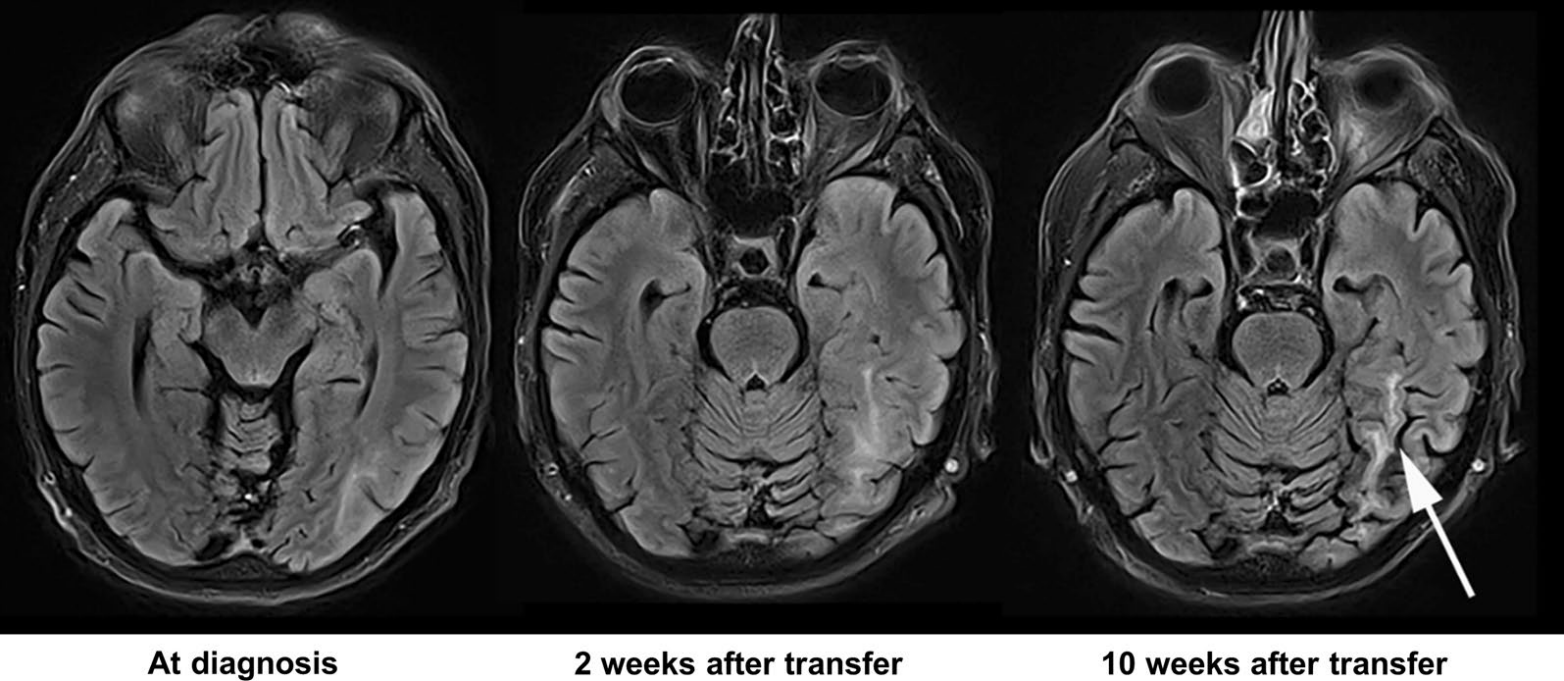
MRI imaging at diagnosis of PML and follow-up. FLAIR MRI at baseline (left) shows a hyperintense T2 lesion in the left temporooccipital region.2 weeks later (middle), regional progression could be noted and PML was diagnosed. Follow-up upon completion of therapy (right) showed containment of lesion extension and regional atrophy (right image, arrow) along with complete regression of contrast agent extravasation (not shown)

In this situation, we stopped multimodal myeloma therapy and initiated treatment with cidofovir and mirtazapine as these drugs have shown some PML activity in anecdotal reports [10, 11]. Furthermore, we administered the last remaining dose of unselected donor lymphocytes containing 1,09 x10^7^ CD3 positive cells/kg body weight to facilitate immune reconstitution. Unfortunately, the clinical presentation did not improve and the patient suffered from recurrent focal seizures with subsequent generalization, progressive symptoms of cortical blindness such as fixation issues and restrictions in the ability to perceive unmoving objects as well as hearing impairment. These clinical findings are consistent with occipital PML involvement documented in MRI scans (Fig. 2).

## Methods

**Fig. 3:**
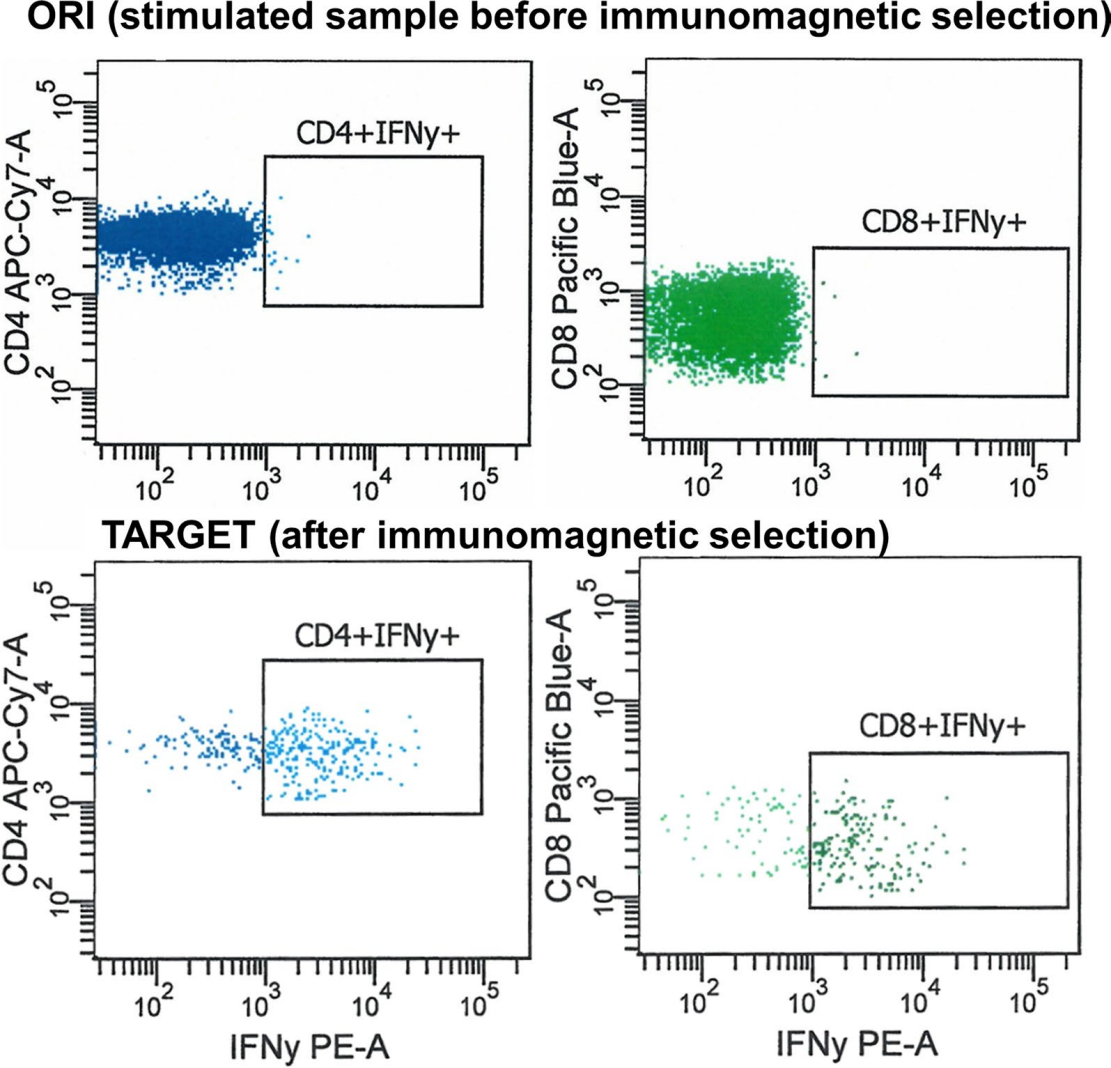
Enrichment of JC-targeted T cells. Top: After stimulation with five non-overlapping JC virus peptide libraries (after stimulation, before selection: ORI), cells were stained with anti-CD45 (FITC), anti-CD3 (APC), anti-CD4 (APC/Cy7), anti-CD8 (PACBlue) and anti-IFNγ (PE), washed and resuspended in acquisition buffer containing 7-AAD. Note a minimal frequency of IFNγ + CD4 + and CD8 + T-cells, yet the absence of IFNγ-bright T-cells. Bottom: Final product (TARGET), after immunomagnetic selection of ORI with anti-IFNγ immunomagnetic beads. JC virus + T-cells are recognized as 7AAD-/CD45bright/SSClow/CD3 + cells with IFNγ on the surface. Enrichment of JC-virus specific T-cells to 65% among CD4 + and 68% among CD8 + T-cells is observed

In an attempt to optimize therapy specificity, we collected lymphocytes from the stem cell donor, an HLA-identical, anti-JC virus-antibody positive family donor by unstimulated peripheral blood leukocyte apheresis [1] to generate a JC virus specific T-cell product. Notably, the donor provided very low numbers of JC virus reactive T cells (Fig. 3). One billion (10^9^) leukocytes, 45% of which were T-cells, served as starting material for selection.

JC virus specific T cells were isolated using the cytokine capture system (CCS) technology. It permits the enrichment of interferon gamma (IFNy) secreting CD4 + and CD8 + T cells. For this, mononuclear cells were labeled with a bispecific anti-CD45/anti-IFNγ antibody. They constitute the catch matrix. We then incubated the yet unselected leukocytes with five overlapping JC virus peptide libraries (LT, ST, VP1, 2 and 3, Miltenyi Biotech, Bergisch-Gladbach, Germany). The cells that can recognize the peptides consequently secrete IFNγ, which is then immobilized by the bispecific CD45/anti-IFNy antibody on the cell surface. We considered those JC virus specific. A second superparamagnetic bead-conjugated anti-IFNγ-antibody marks the cells for subsequent immunomagnetic purification (Fig. 3).

Further materials used were a CliniMACS Prodigy CCS device (Miltenyi Biotec, Bergisch-Gladbach, Germany), a TS500 tubing set and software version 1.2.0.3148, as described in [12]. cGMP conditions were followed throughout and formally validated quality assays were used for release testing in accordance with the manufacturing permit.

The final product contained a 3:2 mix of CD4 + and CD8 + T-cells, two-thirds of each interferon gamma (IFNγ)-positive (Fig. 3), for a total of 20 000 antigen-specific T cells and a dose of potentially allo-reactive, not JC virus responsive T cells of < 200/kg body weight.

## Results

The adoptive cell transfer of JC virus specific T cells was performed without complication roughly 2 months after PML diagnosis. Following these therapies, our patient slowly went into remission of PML with JC virus negative CSF 2.5 months after initial symptoms. Consequently, no further deterioration of vision disorder or sensorineural hearing occurred. We observed containment of PML lesion expansion along with complete regression of contrast agent extravasation 1 month after adoptive transfer (Fig. 2). The observed radiological time course with initial progression and then regional containment during therapy further confirm successful PML therapy, although possibly accentuated by concomitant Immune Reconstitution Inflammatory Syndrome (IRIS). This, however, is an observation rarely made with MRI because the prognosis of PML is typically dismal due to limited treatment options.

Lymphocyte differentiation by immunophenotyping showed NK-cell recovery with frequencies between 20 and 60% (Additional file 1: Table S1). To monitor frequencies of JC virus specific T cells, we repeatedly collected CSF, which remained cell and virus free for multiple examinations at a lower assay sensitivity of 20 copies/dL. Notably, CMV reactivation as well as HPV activity persisted, the latter highlighted by condyloma acuminatum growth and secondary carcinoma development, ultimately requiring surgical intervention 9 months after adaptive JC virus specific T-cell transfer.

Unfortunately, the patient complained about new back pain and the M protein increased during the follow-up period. An FDG PET/CT revealed multiple hypermetabolic lesions throughout the axial skeleton 8 months after PML diagnosis, and we had to resume myeloma therapy. We selected elotuzumab, carfilzomib, lenalidomide, and dexamethasone as salvage regimen to which the patient responded well with very good partial remission (VGPR). Now, 12 months after initial manifestation, focal epilepsy persisted despite antiepileptic therapy, a common neurologic sequelae in PML survivors [13]. However, the patient remains in remission for both PML and myeloma.

## Discussion

We report successful treatment of PML after allo-HCT, using a multimodal approach including withdrawal of immunosuppressive therapy and the adoptive transfer of JC virus specific donor lymphocytes, using the CCS technology. Notably, the donor’s JC virus reactive T cells were barely discernible at the level of detection of our initial flow cytometry assay. Had we screened for JC virus reactive T cells, we would have called this donor negative, as our assay is only validated to frequencies of 1:2 000 T cells or better. Assuming recoveries as previously observed with CMV specific T cells with this technology [12], albeit there for processes with a much greater frequency of targetable cells, the frequency of JC virus reactive T cells in this donor is estimated in the range of 1:10 000. Still, we were able to generate a product of reasonable purity and potentially meaningful cell dose (Fig. 3).

Our observations support a pivotal role of T-cell therapies and are in line with recent case reports on third-party BK virus specific T cells [4] or JC virus specific T cell transfer [5]. Reportedly, several hundred patients have already received CCS T-cells, albeit not for JC virus but for CMV, EBV and AdV [14]. However, the number of treated patients is still very limited. Larger studies comparing these treatments in a randomized fashion will be needed to definitively conclude on the value of JC virus directed T-cell therapies. Moreover, others and we have not been able to demonstrate JC virus specific T-cell expansion at the lesion site as this is difficult and would require brain biopsies.

To the best of our knowledge, this is the first report on PML occurring after Dara-containing combination therapy. We appreciate that our patient was heavily pretreated and post allo-HCT before Dara-containing therapy, which itself put him at risk for PML. An increased risk for reactivated latent infections such as herpes zoster, herpes encephalitis, or hepatitis B during Dara therapy has been described previously and, at least partly, can be attributed to the depletion of NK cells [6]. Possibly, Dara has introduced another type of immunological sequelae highlighted not only by the development of PML but also by concomitant condyloma acuminatum and CMV reactivation. However, the interplay between innate immunity and T-cell responses during CD38 antibody therapy has yet to be elucidated, and the same holds true for Dara treatment post allo-HCT [6].

## Conclusion

In summary, we demonstrate feasibility for generating JC virus specific T cells from a seropositive family donor. Finally, we present the unusual case for successful treatment of PML after allo-HCT, supporting recent reports on virus specific T-cell therapy.

## Supplementary information

**Additional file 1: Table S1.** Immune cell count in peripheral blood during the course of the disease.

## Data Availability

All data and materials, if not given in this article, are openly available on request. All materials used are commercially available.

## Abbreviations

7-AAD: 7-Aminoactinomycin D
AdV: Adenovirus
Allo-HCT: Allogeneic hematopoietic cell transplantation
APC: AlloPhycoCyanin
CCS: Cytokine capture system
CD: Cluster of differentiation
CMV: CytoMegaly virus
CSF: Cerebrospinal fluid
CT: Computed tomography
CR: complete remission
Cy7: Cyanine7
Dara: Daratumumab
DLI: Donor lymphocyte infusion
EBV: Epstein-Barr-Virus
FDG-PET: FluorDesoxyGlucose Positron Emission Tomography
FITC: Fluorescein IsoThioCyanate
FLAIR: FLuid-attenuated inversion recovery
HD: High dose
HPV: Human papilloma virus
IFNy: InterFeroN gamma
IRIS: Immune reconstitution inflammatory syndrome
JCV: John cunningham virus
MM: Multiple myeloma
MRI: Magnetic resonance imaging
NK cells: Natural killer cells
PE: Phycoerythrin
Pom: Pomalidomide
PML: Progressive multifocal leukoencephalopathy
sCR: Stringent complete remission
VGPR: Very good partial remission
